# 2-(4-Fluoro­phen­yl)-1-(4-methyl­phen­yl)-1*H*-phenanthro[9,10-*d*]imidazole

**DOI:** 10.1107/S1600536812037592

**Published:** 2012-09-08

**Authors:** S. Rosepriya, A. Thiruvalluvar, R. Sathishkumar, J. Jayabharathi, Sema Öztürk Yildirim, R.J. Butcher

**Affiliations:** a PG Research Department of Physics, Rajah Serfoji Government College (Autonomous), Thanjavur 613 005, Tamilnadu, India; b Department of Chemistry, Annamalai University, Annamalai Nagar 608 002, Tamilnadu, India; c Department of Chemistry, Howard University, 525 College Street NW, Washington, DC 20059, USA; d Department of Physics, Faculty of Sciences, Erciyes University, 38039 Kayseri, Turkey

## Abstract

The phenanthrene tricyclic ring system in the title mol­ecule, C_28_H_19_FN_2_, is slightly skewed with a dihedral angle of 7.50 (6)° between the outer benzene rings. The *p*-tolyl and fluoro­benzene rings are twisted from the attached imidazole ring by 70.40 (7) and 28.33 (7)°, respectively. In the crystal, C—H⋯F hydrogen bonds link the mol­ecules into zigzag chains in [001], and weak C—H⋯π inter­actions further consolidate the crystal packing.

## Related literature

For related structures, see: Yuan *et al.* (2011 ▶); Rosepriya *et al.* (2011 ▶).
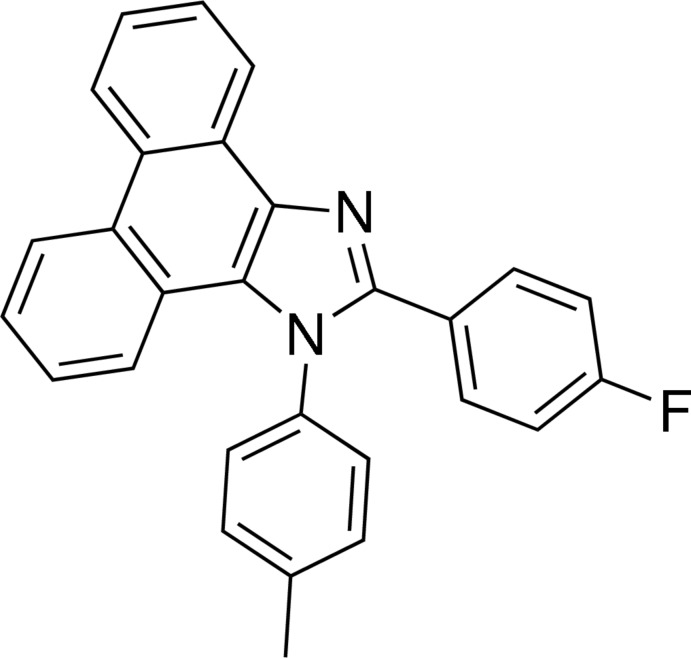


## Experimental

### 

#### Crystal data

C_28_H_19_FN_2_
*M* *_r_* = 402.45
Monoclinic, 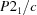

*a* = 10.1809 (2) Å
*b* = 10.7654 (2) Å
*c* = 18.4871 (3) Å
β = 96.115 (1)°
*V* = 2014.68 (6) Å^3^
*Z* = 4
Cu *K*α radiation
μ = 0.67 mm^−1^
*T* = 123 K
0.45 × 0.35 × 0.25 mm

#### Data collection

Agilent Xcalibur Ruby Gemini diffractometer
Absorption correction: multi-scan (*CrysAlis PRO*; Agilent, 2012 ▶) *T*_min_ = 0.753, *T*_max_ = 0.850
8155 measured reflections
4054 independent reflections
3619 reflections with *I* > 2σ(*I*)
*R*_int_ = 0.022

#### Refinement

*R*[*F* ^2^ > 2σ(*F* ^2^)] = 0.040
*wR*(*F* ^2^) = 0.106
*S* = 1.03
4054 reflections
281 parameters
H-atom parameters constrained
Δρ_max_ = 0.25 e Å^−3^
Δρ_min_ = −0.22 e Å^−3^

### 

Data collection: *CrysAlis PRO* (Agilent, 2012 ▶); cell refinement: *CrysAlis PRO*; data reduction: *CrysAlis PRO*; program(s) used to solve structure: *SIR2011* (Burla *et al.*, 2012 ▶); program(s) used to refine structure: *SHELXL97* (Sheldrick, 2008 ▶); molecular graphics: *ORTEP-3* (Farrugia, 1997 ▶) and *PLATON* (Spek, 2009 ▶); software used to prepare material for publication: *SHELXL97*.

## Supplementary Material

Crystal structure: contains datablock(s) global, I. DOI: 10.1107/S1600536812037592/cv5332sup1.cif

Structure factors: contains datablock(s) I. DOI: 10.1107/S1600536812037592/cv5332Isup2.hkl

Supplementary material file. DOI: 10.1107/S1600536812037592/cv5332Isup3.cdx

Additional supplementary materials:  crystallographic information; 3D view; checkCIF report

## Figures and Tables

**Table 1:** Hydrogen-bond geometry (Å, °) *Cg*1, *Cg*2, *Cg*3 and *Cg*4 are the centroids of the N1/C2/N3/C4/C5, C4–C6/C11/C12/C17, C12–C17 and C24–C29 rings, respectively.

*D*—H⋯*A*	*D*—H	H⋯*A*	*D*⋯*A*	*D*—H⋯*A*
C7—H7⋯F4^i^	0.93	2.54	3.1371 (16)	122
C14—H14⋯*Cg*2^ii^	0.93	2.95	3.5613 (15)	125
C20—H20⋯*Cg*2^iii^	0.93	2.84	3.4540 (14)	125
C23—H23⋯*Cg*3^iv^	0.93	2.81	3.5709 (14)	140
C30—H30*A*⋯*Cg*1^iii^	0.96	2.91	3.4294 (17)	115
C30—H30*B*⋯*Cg*4^i^	0.96	2.77	3.6659 (16)	155

Symmetry codes: (i) 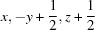; (ii) 
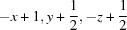
; (iii) 
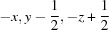
; (iv) 
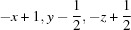
.

**Fig. 1.**
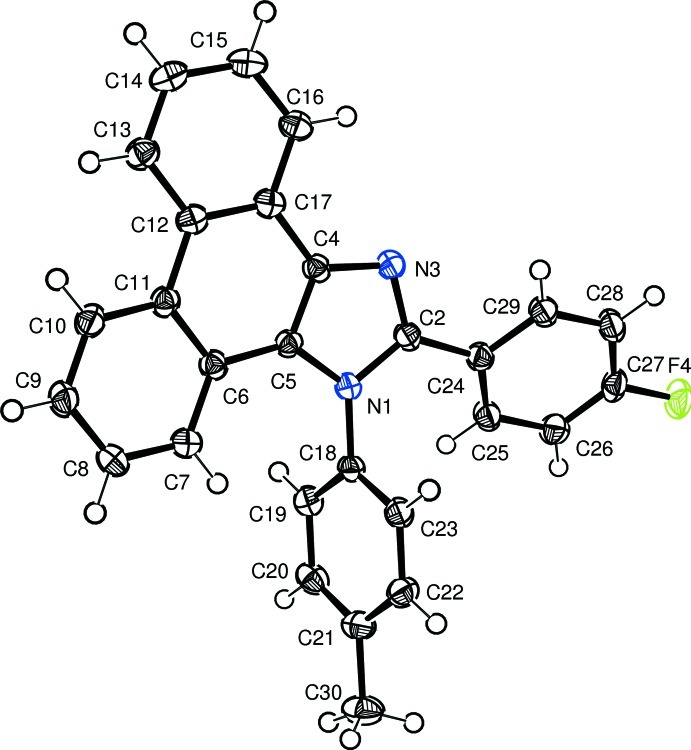
The molecular structure of (I), with displacement ellipsoids drawn at the 50% probability level. H atoms are shown as small spheres of arbitrary radius.

**Fig. 2.**
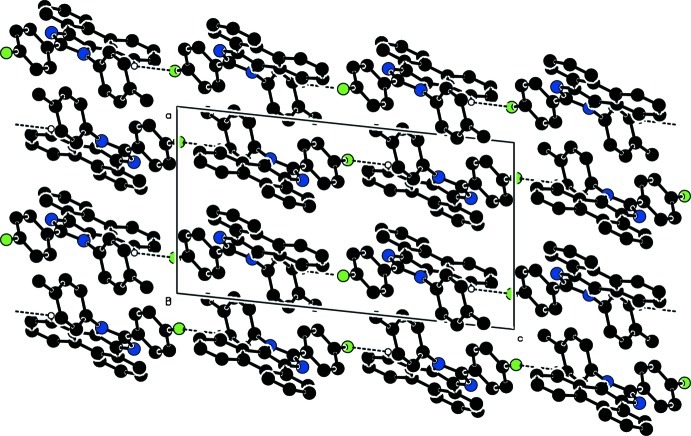
A portion of the crystal packing viewed down the b axis. Dashed lines indicate C—H···F hydrogen bonds. H atoms not involved in hydrogen bonding have been omitted.

## supplementary crystallographic information

### Comment

Yuan *et al.* (2011) have reported synthesis, single-crystal structures, photophysical, electrochemical and mobility properties of four phenanthroimidazole derivatives, namely, 1,2-diphenyl-1*H*-phenanthro[9,10 -d]imidazole, 2-phenyl-1-*p*-tolyl-1*H*-phenanthro[9,10 -d]imidazole, 1-phenyl-2-*p*-tolyl-1*H*-phenanthro-[9,10 -d]imidazole and 1,2-di-*p*-tolyl-1*H*-phenanthro[9,10 -d]imidazole. Since our research group deals with the hole transport materials and organic light emitting devices, we are interested in the title compound, (I), as a ligand for Iridium complexes.

In (I) (Fig. 1), all bond lengths and angles are normal and correspond to those observed in the related compounds (Yuan *et al.*, 2011; Rosepriya *et al.*, 2011). The phenanthrene tricycle is stranded with a dihedral angle of 7.50 (6)° between the utmost benzene rings. The *p*-tolyl and fluorobenzene rings are twisted from the attached imidazole ring at 70.40 (7) and 28.33 (7)°, respectively. The dihedral angle between the *p*-tolyl and fluorobenzene rings is 66.63 (6) °.

In the crystal, C7—H7···F4 hydrogen bonds (Table 1) link the molecules into zigzag chains in [001] (Fig. 2), and weak C—H···π interactions (Table 1) consolidate further the crystal packing.

### Experimental

The title compound has been synthesized by a mixture of phenanthrene-9,10-dione (1.0 g, 4.8 mmol), ammonium acetate (1.48 g, 19.2 mmol), 4-fluorobenzaldehyde (0.6 g, 4.3 mmol) and 4-methylaniline (2.95 g, 24 mmol). These four components have been refluxed in ethanol (20 ml) at 353 K. The reaction was monitored by TLC and purified by column chromatography using petroleum ether: ethyl acetate (9:1) as the eluent. Yield: 0.8 g (50%). The compound was dissolved in dimethyl sulfoxide and allowed to slow evaporation, to obtain crystals suitable for X-ray diffraction studies.

### Refinement

The H atoms were positioned geometrically and allowed to ride on their parent atoms, with C—H = 0.93 and 0.96 Å for C*sp*^2^ and methyl H atoms, respectively. *U*_iso_(H) = *xU*_eq_(C), where *x* = 1.5 for methyl H atoms and 1.2 for other C-bound H atoms.

### Crystal data

**Table:**

C_28_H_19_FN_2_	*F*(000) = 840
*M**_r_* = 402.45	*D*_x_ = 1.327 Mg m^−^^3^
Monoclinic, *P*2_1_/*c*	Melting point: 494 K
Hall symbol: -P 2ybc	Cu *K*α radiation, λ = 1.54184 Å
*a* = 10.1809 (2) Å	Cell parameters from 4975 reflections
*b* = 10.7654 (2) Å	θ = 4.1–75.3°
*c* = 18.4871 (3) Å	µ = 0.67 mm^−^^1^
β = 96.115 (1)°	*T* = 123 K
*V* = 2014.68 (6) Å^3^	Block, colourless
*Z* = 4	0.45 × 0.35 × 0.25 mm

### Data collection

**Table:**

Agilent Xcalibur Ruby Gemini diffractometer	4054 independent reflections
Radiation source: Enhance (Cu) X-ray Source	3619 reflections with *I* > 2σ(*I*)
Graphite monochromator	*R*_int_ = 0.022
Detector resolution: 10.5081 pixels mm^-1^	θ_max_ = 75.5°, θ_min_ = 4.4°
ω scans	*h* = −11→12
Absorption correction: multi-scan (*CrysAlis PRO*; Agilent, 2012)	*k* = −11→13
*T*_min_ = 0.753, *T*_max_ = 0.850	*l* = −22→13
8155 measured reflections	

### Refinement

**Table:**

Refinement on *F*^2^	Primary atom site location: structure-invariant direct methods
Least-squares matrix: full	Secondary atom site location: difference Fourier map
*R*[*F*^2^ > 2σ(*F*^2^)] = 0.040	Hydrogen site location: inferred from neighbouring sites
*wR*(*F*^2^) = 0.106	H-atom parameters constrained
*S* = 1.03	*w* = 1/[σ^2^(*F*_o_^2^) + (0.0517*P*)^2^ + 0.6604*P*] where *P* = (*F*_o_^2^ + 2*F*_c_^2^)/3
4054 reflections	(Δ/σ)_max_ = 0.001
281 parameters	Δρ_max_ = 0.25 e Å^−^^3^
0 restraints	Δρ_min_ = −0.22 e Å^−^^3^

### Special details

**Table:**

Geometry. Bond distances, angles *etc*. have been calculated using the rounded fractional coordinates. All su's are estimated from the variances of the (full) variance-covariance matrix. The cell e.s.d.'s are taken into account in the estimation of distances, angles and torsion angles
Refinement. Refinement of *F*^2^ against ALL reflections. The weighted *R*-factor *wR* and goodness of fit *S* are based on *F*^2^, conventional *R*-factors *R* are based on *F*, with *F* set to zero for negative *F*^2^. The threshold expression of *F*^2^ > 2σ(*F*^2^) is used only for calculating *R*-factors(gt) *etc*. and is not relevant to the choice of reflections for refinement. *R*-factors based on *F*^2^ are statistically about twice as large as those based on *F*, and *R*- factors based on ALL data will be even larger.

### Fractional atomic coordinates and isotropic or equivalent isotropic displacement parameters (Å^2^)

**Table:**

	*x*	*y*	*z*	*U*_iso_*/*U*_eq_	
F4	0.18868 (10)	0.04100 (8)	−0.00640 (5)	0.0413 (3)	
N1	0.22790 (10)	0.50374 (10)	0.22352 (5)	0.0213 (3)	
N3	0.32302 (10)	0.57512 (10)	0.12764 (6)	0.0230 (3)	
C2	0.26496 (12)	0.47886 (12)	0.15502 (7)	0.0222 (3)	
C4	0.32401 (12)	0.66606 (12)	0.18005 (6)	0.0213 (3)	
C5	0.26743 (12)	0.62474 (11)	0.24026 (6)	0.0207 (3)	
C6	0.26588 (12)	0.69804 (12)	0.30544 (6)	0.0212 (3)	
C7	0.22497 (13)	0.65420 (12)	0.37131 (7)	0.0253 (3)	
C8	0.23298 (14)	0.72837 (13)	0.43239 (7)	0.0282 (4)	
C9	0.28381 (14)	0.84865 (13)	0.43030 (7)	0.0299 (4)	
C10	0.32612 (13)	0.89271 (13)	0.36685 (7)	0.0273 (4)	
C11	0.31801 (12)	0.82110 (12)	0.30268 (7)	0.0223 (3)	
C12	0.36582 (12)	0.86934 (12)	0.23605 (7)	0.0227 (3)	
C13	0.40522 (13)	0.99443 (13)	0.22925 (7)	0.0266 (4)	
C14	0.44945 (13)	1.03779 (13)	0.16616 (8)	0.0296 (4)	
C15	0.45837 (13)	0.95801 (14)	0.10720 (7)	0.0298 (4)	
C16	0.41987 (13)	0.83594 (13)	0.11144 (7)	0.0260 (4)	
C17	0.37198 (12)	0.79098 (12)	0.17515 (7)	0.0221 (3)	
C18	0.17293 (12)	0.41523 (12)	0.27015 (6)	0.0214 (3)	
C19	0.04292 (13)	0.42709 (13)	0.28571 (7)	0.0258 (4)	
C20	−0.00771 (13)	0.34031 (13)	0.33123 (7)	0.0287 (4)	
C21	0.06862 (14)	0.24164 (13)	0.36048 (7)	0.0286 (4)	
C22	0.19820 (14)	0.23184 (13)	0.34311 (7)	0.0286 (4)	
C23	0.25117 (13)	0.31826 (12)	0.29870 (7)	0.0247 (3)	
C24	0.24119 (12)	0.36103 (12)	0.11511 (7)	0.0231 (3)	
C25	0.13499 (13)	0.28159 (13)	0.12329 (7)	0.0276 (4)	
C26	0.11766 (14)	0.17314 (13)	0.08261 (7)	0.0299 (4)	
C27	0.20587 (15)	0.14662 (13)	0.03352 (7)	0.0291 (4)	
C28	0.31057 (14)	0.22303 (13)	0.02274 (7)	0.0297 (4)	
C29	0.32810 (13)	0.33064 (13)	0.06400 (7)	0.0263 (4)	
C30	0.01479 (16)	0.14695 (15)	0.41001 (8)	0.0383 (4)	
H7	0.19202	0.57389	0.37360	0.0303*	
H8	0.20442	0.69819	0.47518	0.0339*	
H9	0.28904	0.89866	0.47152	0.0358*	
H10	0.36123	0.97241	0.36629	0.0327*	
H13	0.40115	1.04845	0.26821	0.0319*	
H14	0.47357	1.12073	0.16270	0.0355*	
H15	0.49032	0.98744	0.06514	0.0357*	
H16	0.42550	0.78302	0.07213	0.0312*	
H19	−0.00949	0.49204	0.26601	0.0310*	
H20	−0.09428	0.34849	0.34230	0.0344*	
H22	0.25028	0.16587	0.36171	0.0343*	
H23	0.33821	0.31109	0.28827	0.0296*	
H25	0.07540	0.30157	0.15628	0.0331*	
H26	0.04784	0.11968	0.08852	0.0358*	
H28	0.36809	0.20301	−0.01134	0.0356*	
H29	0.39843	0.38321	0.05761	0.0315*	
H30A	−0.07357	0.16950	0.41836	0.0575*	
H30B	0.06969	0.14470	0.45555	0.0575*	
H30C	0.01404	0.06650	0.38757	0.0575*	

### Atomic displacement parameters (Å^2^)

**Table:**

	*U*^11^	*U*^22^	*U*^33^	*U*^12^	*U*^13^	*U*^23^
F4	0.0566 (6)	0.0297 (4)	0.0377 (5)	0.0015 (4)	0.0052 (4)	−0.0148 (4)
N1	0.0245 (5)	0.0200 (5)	0.0198 (5)	−0.0001 (4)	0.0042 (4)	−0.0014 (4)
N3	0.0239 (5)	0.0235 (5)	0.0219 (5)	0.0008 (4)	0.0038 (4)	−0.0008 (4)
C2	0.0226 (6)	0.0242 (6)	0.0201 (6)	0.0027 (5)	0.0037 (4)	−0.0003 (5)
C4	0.0213 (5)	0.0230 (6)	0.0196 (5)	0.0018 (5)	0.0023 (4)	0.0005 (5)
C5	0.0209 (5)	0.0198 (6)	0.0213 (6)	0.0009 (5)	0.0020 (4)	0.0005 (5)
C6	0.0216 (5)	0.0219 (6)	0.0202 (6)	0.0023 (5)	0.0021 (4)	−0.0002 (5)
C7	0.0284 (6)	0.0237 (6)	0.0241 (6)	−0.0009 (5)	0.0049 (5)	0.0008 (5)
C8	0.0342 (7)	0.0306 (7)	0.0206 (6)	0.0003 (5)	0.0063 (5)	−0.0007 (5)
C9	0.0395 (7)	0.0275 (7)	0.0225 (6)	0.0023 (6)	0.0030 (5)	−0.0052 (5)
C10	0.0330 (7)	0.0211 (6)	0.0276 (6)	−0.0001 (5)	0.0024 (5)	−0.0022 (5)
C11	0.0226 (6)	0.0213 (6)	0.0227 (6)	0.0026 (5)	0.0013 (4)	0.0002 (5)
C12	0.0207 (5)	0.0228 (6)	0.0241 (6)	0.0002 (5)	0.0005 (4)	0.0023 (5)
C13	0.0274 (6)	0.0245 (7)	0.0275 (6)	−0.0026 (5)	0.0009 (5)	−0.0001 (5)
C14	0.0289 (6)	0.0247 (7)	0.0344 (7)	−0.0052 (5)	−0.0002 (5)	0.0066 (6)
C15	0.0282 (7)	0.0346 (7)	0.0266 (6)	−0.0038 (5)	0.0033 (5)	0.0086 (6)
C16	0.0243 (6)	0.0305 (7)	0.0230 (6)	−0.0003 (5)	0.0021 (5)	0.0028 (5)
C17	0.0189 (5)	0.0245 (6)	0.0226 (6)	0.0012 (5)	0.0011 (4)	0.0025 (5)
C18	0.0258 (6)	0.0204 (6)	0.0184 (5)	−0.0032 (5)	0.0039 (4)	−0.0021 (5)
C19	0.0253 (6)	0.0271 (7)	0.0251 (6)	0.0011 (5)	0.0027 (5)	−0.0011 (5)
C20	0.0241 (6)	0.0356 (7)	0.0270 (6)	−0.0064 (5)	0.0054 (5)	−0.0034 (6)
C21	0.0352 (7)	0.0285 (7)	0.0218 (6)	−0.0104 (5)	0.0021 (5)	−0.0013 (5)
C22	0.0350 (7)	0.0238 (7)	0.0267 (6)	0.0010 (5)	0.0017 (5)	0.0019 (5)
C23	0.0255 (6)	0.0251 (6)	0.0239 (6)	0.0002 (5)	0.0045 (5)	−0.0021 (5)
C24	0.0264 (6)	0.0230 (6)	0.0198 (6)	0.0028 (5)	0.0017 (4)	−0.0002 (5)
C25	0.0295 (6)	0.0286 (7)	0.0252 (6)	−0.0005 (5)	0.0057 (5)	−0.0048 (5)
C26	0.0338 (7)	0.0268 (7)	0.0289 (7)	−0.0032 (5)	0.0029 (5)	−0.0023 (5)
C27	0.0399 (7)	0.0221 (6)	0.0242 (6)	0.0051 (5)	−0.0013 (5)	−0.0058 (5)
C28	0.0345 (7)	0.0316 (7)	0.0237 (6)	0.0083 (6)	0.0066 (5)	−0.0030 (5)
C29	0.0274 (6)	0.0275 (7)	0.0241 (6)	0.0027 (5)	0.0039 (5)	0.0000 (5)
C30	0.0428 (8)	0.0407 (8)	0.0313 (7)	−0.0165 (7)	0.0031 (6)	0.0049 (6)

### Geometric parameters (Å, º)

**Table:**

F4—C27	1.3567 (16)	C21—C30	1.512 (2)
N1—C2	1.3855 (16)	C22—C23	1.3874 (19)
N1—C5	1.3885 (16)	C24—C25	1.3993 (18)
N1—C18	1.4377 (16)	C24—C29	1.4003 (18)
N3—C2	1.3203 (17)	C25—C26	1.3898 (19)
N3—C4	1.3767 (16)	C26—C27	1.373 (2)
C2—C24	1.4744 (18)	C27—C28	1.378 (2)
C4—C5	1.3803 (16)	C28—C29	1.3881 (19)
C4—C17	1.4369 (18)	C7—H7	0.9300
C5—C6	1.4419 (16)	C8—H8	0.9300
C6—C7	1.4097 (17)	C9—H9	0.9300
C6—C11	1.4301 (18)	C10—H10	0.9300
C7—C8	1.3783 (18)	C13—H13	0.9300
C8—C9	1.397 (2)	C14—H14	0.9300
C9—C10	1.3763 (19)	C15—H15	0.9300
C10—C11	1.4098 (18)	C16—H16	0.9300
C11—C12	1.4669 (18)	C19—H19	0.9300
C12—C13	1.4145 (19)	C20—H20	0.9300
C12—C17	1.4136 (18)	C22—H22	0.9300
C13—C14	1.3758 (19)	C23—H23	0.9300
C14—C15	1.398 (2)	C25—H25	0.9300
C15—C16	1.376 (2)	C26—H26	0.9300
C16—C17	1.4077 (18)	C28—H28	0.9300
C18—C19	1.3900 (18)	C29—H29	0.9300
C18—C23	1.3832 (18)	C30—H30A	0.9600
C19—C20	1.3925 (19)	C30—H30B	0.9600
C20—C21	1.391 (2)	C30—H30C	0.9600
C21—C22	1.395 (2)		
			
C2—N1—C5	106.52 (10)	C24—C25—C26	120.66 (12)
C2—N1—C18	125.54 (10)	C25—C26—C27	118.56 (13)
C5—N1—C18	127.60 (10)	F4—C27—C26	118.57 (13)
C2—N3—C4	104.97 (11)	F4—C27—C28	118.60 (12)
N1—C2—N3	112.04 (11)	C26—C27—C28	122.83 (13)
N1—C2—C24	125.28 (11)	C27—C28—C29	118.36 (13)
N3—C2—C24	122.66 (11)	C24—C29—C28	120.82 (12)
N3—C4—C5	111.47 (11)	C6—C7—H7	119.00
N3—C4—C17	126.74 (11)	C8—C7—H7	119.00
C5—C4—C17	121.76 (11)	C7—C8—H8	120.00
N1—C5—C4	104.98 (10)	C9—C8—H8	120.00
N1—C5—C6	132.14 (11)	C8—C9—H9	120.00
C4—C5—C6	122.70 (11)	C10—C9—H9	120.00
C5—C6—C7	124.70 (12)	C9—C10—H10	119.00
C5—C6—C11	116.11 (10)	C11—C10—H10	119.00
C7—C6—C11	119.07 (11)	C12—C13—H13	119.00
C6—C7—C8	121.11 (12)	C14—C13—H13	119.00
C7—C8—C9	120.28 (12)	C13—C14—H14	120.00
C8—C9—C10	119.58 (12)	C15—C14—H14	120.00
C9—C10—C11	122.22 (13)	C14—C15—H15	120.00
C6—C11—C10	117.73 (11)	C16—C15—H15	120.00
C6—C11—C12	121.03 (11)	C15—C16—H16	120.00
C10—C11—C12	121.21 (12)	C17—C16—H16	120.00
C11—C12—C13	122.30 (12)	C18—C19—H19	120.00
C11—C12—C17	120.29 (11)	C20—C19—H19	120.00
C13—C12—C17	117.41 (12)	C19—C20—H20	119.00
C12—C13—C14	121.32 (12)	C21—C20—H20	119.00
C13—C14—C15	120.45 (13)	C21—C22—H22	119.00
C14—C15—C16	119.98 (12)	C23—C22—H22	119.00
C15—C16—C17	120.15 (12)	C18—C23—H23	120.00
C4—C17—C12	117.65 (11)	C22—C23—H23	120.00
C4—C17—C16	121.66 (12)	C24—C25—H25	120.00
C12—C17—C16	120.66 (12)	C26—C25—H25	120.00
N1—C18—C19	120.22 (11)	C25—C26—H26	121.00
N1—C18—C23	118.94 (11)	C27—C26—H26	121.00
C19—C18—C23	120.85 (12)	C27—C28—H28	121.00
C18—C19—C20	119.04 (12)	C29—C28—H28	121.00
C19—C20—C21	121.32 (12)	C24—C29—H29	120.00
C20—C21—C22	118.12 (12)	C28—C29—H29	120.00
C20—C21—C30	121.79 (13)	C21—C30—H30A	109.00
C22—C21—C30	120.09 (13)	C21—C30—H30B	109.00
C21—C22—C23	121.49 (13)	C21—C30—H30C	109.00
C18—C23—C22	119.18 (12)	H30A—C30—H30B	109.00
C2—C24—C25	124.00 (11)	H30A—C30—H30C	109.00
C2—C24—C29	117.18 (11)	H30B—C30—H30C	109.00
C25—C24—C29	118.76 (12)		
			
C5—N1—C2—N3	−0.67 (14)	C7—C8—C9—C10	−0.1 (2)
C5—N1—C2—C24	−179.32 (12)	C8—C9—C10—C11	1.1 (2)
C18—N1—C2—N3	−174.41 (11)	C9—C10—C11—C6	−1.14 (19)
C18—N1—C2—C24	6.95 (19)	C9—C10—C11—C12	−178.96 (13)
C2—N1—C5—C4	1.00 (13)	C6—C11—C12—C13	173.38 (12)
C2—N1—C5—C6	−174.12 (13)	C6—C11—C12—C17	−5.61 (18)
C18—N1—C5—C4	174.57 (11)	C10—C11—C12—C13	−8.88 (19)
C18—N1—C5—C6	−0.6 (2)	C10—C11—C12—C17	172.14 (12)
C2—N1—C18—C19	−112.80 (14)	C11—C12—C13—C14	−179.83 (12)
C2—N1—C18—C23	66.83 (16)	C17—C12—C13—C14	−0.82 (19)
C5—N1—C18—C19	74.79 (16)	C11—C12—C17—C4	3.00 (18)
C5—N1—C18—C23	−105.58 (15)	C11—C12—C17—C16	−178.90 (12)
C4—N3—C2—N1	0.03 (13)	C13—C12—C17—C4	−176.03 (11)
C4—N3—C2—C24	178.71 (11)	C13—C12—C17—C16	2.07 (18)
C2—N3—C4—C5	0.65 (14)	C12—C13—C14—C15	−1.0 (2)
C2—N3—C4—C17	−177.29 (12)	C13—C14—C15—C16	1.5 (2)
N1—C2—C24—C25	28.9 (2)	C14—C15—C16—C17	−0.3 (2)
N1—C2—C24—C29	−153.74 (12)	C15—C16—C17—C4	176.46 (12)
N3—C2—C24—C25	−149.60 (13)	C15—C16—C17—C12	−1.56 (19)
N3—C2—C24—C29	27.75 (18)	N1—C18—C19—C20	−179.79 (11)
N3—C4—C5—N1	−1.05 (14)	C23—C18—C19—C20	0.59 (19)
N3—C4—C5—C6	174.66 (11)	N1—C18—C23—C22	−179.33 (11)
C17—C4—C5—N1	177.01 (11)	C19—C18—C23—C22	0.30 (19)
C17—C4—C5—C6	−7.28 (19)	C18—C19—C20—C21	−0.8 (2)
N3—C4—C17—C12	−178.97 (12)	C19—C20—C21—C22	0.2 (2)
N3—C4—C17—C16	2.9 (2)	C19—C20—C21—C30	179.94 (13)
C5—C4—C17—C12	3.28 (18)	C20—C21—C22—C23	0.8 (2)
C5—C4—C17—C16	−174.80 (12)	C30—C21—C22—C23	−179.02 (13)
N1—C5—C6—C7	3.0 (2)	C21—C22—C23—C18	−1.0 (2)
N1—C5—C6—C11	178.89 (12)	C2—C24—C25—C26	178.69 (12)
C4—C5—C6—C7	−171.39 (12)	C29—C24—C25—C26	1.39 (19)
C4—C5—C6—C11	4.49 (18)	C2—C24—C29—C28	−178.28 (12)
C5—C6—C7—C8	176.56 (13)	C25—C24—C29—C28	−0.8 (2)
C11—C6—C7—C8	0.80 (19)	C24—C25—C26—C27	−0.9 (2)
C5—C6—C11—C10	−175.94 (11)	C25—C26—C27—F4	−179.75 (12)
C5—C6—C11—C12	1.88 (17)	C25—C26—C27—C28	−0.2 (2)
C7—C6—C11—C10	0.18 (18)	F4—C27—C28—C29	−179.67 (12)
C7—C6—C11—C12	178.00 (12)	C26—C27—C28—C29	0.8 (2)
C6—C7—C8—C9	−0.9 (2)	C27—C28—C29—C24	−0.3 (2)

### Hydrogen-bond geometry (Å, º)

Cg1, Cg2, Cg3 and Cg4 are the centroids of the N1/C2/N3/C4/C5, C4–C6/C11/C12/C17, C12–C17 and C24–C29 rings, respectively.

**Table:**

*D*—H···*A*	*D*—H	H···*A*	*D*···*A*	*D*—H···*A*
C7—H7···F4^i^	0.93	2.54	3.1371 (16)	122
C14—H14···*Cg*2^ii^	0.93	2.95	3.5613 (15)	125
C20—H20···*Cg*2^iii^	0.93	2.84	3.4540 (14)	125
C23—H23···*Cg*3^iv^	0.93	2.81	3.5709 (14)	140
C30—H30*A*···*Cg*1^iii^	0.96	2.91	3.4294 (17)	115
C30—H30*B*···*Cg*4^i^	0.96	2.77	3.6659 (16)	155

Symmetry codes: (i) *x*, −*y*+1/2, *z*+1/2; (ii) −*x*+1, *y*+1/2, −*z*+1/2; (iii) −*x*, *y*−1/2, −*z*+1/2; (iv) −*x*+1, *y*−1/2, −*z*+1/2.
